# Effects of Resistance Training as a Behavioural Preventive Measure on Musculoskeletal Complaints, Maximum Strength and Ergonomic Risk in Dentists and Dental Assistants

**DOI:** 10.3390/s22208069

**Published:** 2022-10-21

**Authors:** Fabian Holzgreve, Laura Fraeulin, Christian Maurer-Grubinger, Werner Betz, Christina Erbe, Tim Weis, Keno Janssen, Lisa Schulte, Amaya de Boer, Albert Nienhaus, David A. Groneberg, Daniela Ohlendorf

**Affiliations:** 1 Institute of Occupational Medicine, Social Medicine and Environmental Medicine, Goethe University, Building 9a, Theodor-Stern-Kai 7, 60596 Frankfurt am Main, Germany; 2 Institute of Dentistry, Department of Dental Radiology, Goethe University, Theodor-Stern-Kai 7, 60596 Frankfurt am Main, Germany; 3 Department of Orthodontics, University Medical Center of the Johannes Gutenberg-University Mainz, Augustusplatz 2, 55131 Mainz, Germany; 4 Principles of Prevention and Rehabilitation Department (GPR), Institute for Statutory Accident Insurance and Prevention in the Health and Welfare Services (BGW), 22089 Hamburg, Germany

**Keywords:** musculoskeletal diseases, RULA, inertial motion capture, inertial sensors, strength training, Nordic Questionnaire, VAS, kinematic analysis

## Abstract

Introduction: For dental professionals, musculoskeletal disorders (MSD) are common health hazards and resistance training programmes have been promising approaches in the quest for a reduction in the pain intensity of these professionals. Therefore, the aim of the current study was to investigate the effect of a trunk-oriented 10-week resistance training programme. Method: In total, the study was conducted with 17 dentists and dental assistants (3 m/14 f) over a course of 10 weeks, with workouts being performed 2 times a week using a 60 min intervention programme consisting of 11 resistance training exercises. The outcome values that were collected were the pain intensity (visual analogue scale (VAS) combined with a modified version of the Nordic Questionnaire), the MVIC and the rapid upper limb assessment (RULA) score (based on data from inertial motion units) during a standardised dental treatment protocol. Results: A significant reduction in pain intensity was found for each queried body region: the neck, upper back, lower back and the right and left shoulders. The maximum voluntary isometric contraction (MVIC) improved significantly in all outcome measures: flexion, extension, right and left lateral flexion and right and left rotation. Conclusions: A 10-week resistance training programme for dentists and dental assistants had significant effects on pain intensity reduction and the MVIC of the musculature of the trunk and is, therefore, suitable as a behavioural preventive measure against MSD in dental professionals.

## 1. Introduction

Musculoskeletal disorders (MSD) are a common health hazard in dental professionals [1,2,3,4,5,6,7] and often lead to sick leave or even premature ill-health retirement [7]. The most affected body regions are the shoulders, the wrists, the lower and upper back, and the neck [1,4,6,8,9].

One major risk factor for MSD in dentistry is the adoption of awkward static postures, which are maintained during dental treatment [4,9,10]. In their literature review, Valachi et al. [10] state that the static sitting posture, which is characterised by a flexed and, at the same time, laterally bent and rotated trunk, is associated with spinal hypomobility and increased disc pressure, which can eventually lead to injury or low back pain. When treating patients over several hours a day, these asymmetric postures may, in the long term, cause strain on the postural trunk muscles because these muscles have to work in predominantly static contractions at inconvenient muscle lengths. It has been shown that contractions at unfavourable muscle lengths and at awkward moment angles during biaxial movements can decrease the actual back strength by up to 40% [11] or even 56% [12], depending on the plane of motion. This means that the involved muscles cannot access their usual strength potential, which can be exhausting over the long hours of a working day. Eventually, this can cause ischemia and, consequently, lead to imbalances and pain [13].

As a first impulse, a prevention strategy would be to change the working environment by means of the inventory arrangement (arrangement of the operating elements of the dental treatment unit) in order to enable a more favourable posture. However, at the moment, there are no pure dental workplace concepts available that would allow for a more ergonomic working posture during dental treatment. The previous analysis of all four currently available concepts by the Rapid Upper Limb Assessment (RULA) revealed alarming results, highlighting the great urgency for change [14]. Improving the situation for dental professionals by changing the arrangement of the inventory, therefore, seems virtually impossible. Instead, one promising prevention strategy might be to increase the dental professional’s strength potential through regular resistance training [15,16,17]. If the muscles possess a higher strength potential, a lower percentage of the one repetition maximum will eventually be required to hold the upper body in position and, thus, this may enable dentists as well as dental assistants to endure the described postures for longer and be free from pain.

In dentistry, so far, only a pilot cohort study by Yiu et al. [17] has demonstrated that a 10-week resistance training programme (4 progressive exercises for the neck and shoulders; 5 times per week; 2 min per session) can improve the RULA score (1.88; 95% CI 1.05–2.70; *p* < 0.01) as well as the isometric maximal strength in the neck muscles (flexors: 7.2%; 95% CI 6.9–27.6%; *p* < 0.01; extensors: 23.2%; 95% CI 10.2–36.3%; *p* < 0.01), although the pain severity remained unchanged. Whilst pain reduction ought to be the main goal of a prevention strategy, it should be noted that in Yiu et al.’s study [17], the participants were recruited exclusively among students who had low working experience and pain was not an inclusion criterion. In fact, on a 10-point scale, the symptom severity was only 2.6 for the neck and 2.3 for the shoulders. Therefore, the extent of the validity of the intervention is questionable.

This approach, however, is still promising since resistance training has been successfully applied to other occupational settings for reducing MSD in a short period of time [18,19,20,21,22]. For example, Zebis et al. [16] presented a resistance training programme for female office workers that led to a rapid decrease in neck pain intensity during the first 7 weeks of training compared to a control group. Besides the quick benefits, positive long-term effects have also been described; a brief elastic resistance training programme over the course of 10 weeks reduced the neck/shoulder pain in female office workers by 40% compared to a control group [22]. In addition, a 6% increase in isometric strength and prolonged relaxation phases in muscle activity were reported directly after the intervention and also in a follow-up, which was ten weeks after the intervention [22]. Furthermore, positive effects from strength training for the reduction of pain and discomfort showed further encouraging results when training with dumbbells and barbells [18,19,20]. For example, shoulder and neck complaints were successfully reduced over a period of 20 weeks with the help of dynamic strength training 3 times a week for 20 min each in office workers [19]. Analogously, strength training performed 3 times per week for 20 weeks in industrial workers for the upper extremities showed positive effects on forearm discomfort and work disability [18] resistance training with dumbbells also showed positive effects on further health parameters and overall strength [20].

One could speculate that not only does the pain intensity decrease with increasing muscle strength, but the working postures are also possibly improved—as long as optimal sight into the patients’ mouth remains. Once the pain has eased, the adopted protective postures may no longer be needed by the dental personnel.

The aim of the current study was to investigate the effect of a trunk-oriented 10-week resistance training programme that was especially designed for dental professionals regarding: (a) the pain intensity in the upper and lower back, the neck and the left and right shoulders; (b) the isometric muscle strength of the addressed muscles; and (c) working posture.

The current study is part of the SOPEZ project (“study for the optimization of ergonomics in dental practice” [23]).

## 2. Materials & Methods

### 2.1. Subjects

In total, 20 participants volunteered to take part in this intervention study, although 3 participants (3 females) were not able to complete the programme. Seventeen participants (3 males/14 females) finished the programme successfully; of these, 6 (6 females) participants worked as dental assistants and 11 (3 males/8 females) as dentists in Germany. The participants were 35.7 ± 12.4 years old, 169.3 ± 6.3 cm tall, weighed 62.9 ± 8.7 kg and had a BMI of 21.9 ± 2.2 kg/m^2^ (Table 1). Only right-handed participants were included in this study. In this regard, the principal investigator relied on the accuracy of the information provided by the subjects. Moreover, only participants with annual musculoskeletal pain in relevant regions (i.e., the neck, upper back, lower back or shoulders) were eligible to take part in this study. Musculoskeletal pain was queried using a modified Nordic Questionnaire [24] before the study. The acquisition was carried out in two ways. First, subjects participated in a biomechanical analysis as part of the SOPEZ project. Second, flyers were distributed to dental practices in the Frankfurt am Main area. Subjects were instructed not to take pain-relieving medications during the intervention. If they were unable to refrain from taking pain medication, subjects should inform the study administration about the type and dosage of painkillers. All participants had to confirm prior to the study that they had no current injuries in the musculoskeletal system (e.g., urgent herniated discs, slipped vertebra), severely restrictive malformations of the spine, rheumatic diseases or stiffened spinal joints and that any relevant surgery had taken place at least two years previously. This research complied with the tenets of the Declaration of Helsinki. Informed consent was obtained from each participant. This study was approved by the ethics research committee of Goethe University (356/17) in Frankfurt am Main, Germany.

### 2.2. Assessment Tools

- Nordic Questionnaire and Nordic Questionnaire combined with VAS scales

Before the study, the modified Nordic Questionnaire [24] was used as a sample selection tool. Only participants with annual musculoskeletal pain in relevant regions (i.e., the neck, upper back, lower back or shoulders) were eligible to take part in this study.

A different modified version of the Nordic Questionnaire, by which the subjects indicated pain intensity on a visual analogue scale (VAS), was used as an evaluation tool in order to compare the pain intensities before and after the intervention in different body regions. This contains stripes of 10 cm that indicate the pain intensity on a scale of no pain intensity to maximum pain intensity (see Supplementary Material). The validity, reliability and sensitivity to changes in pain have been rated as high using this method [25,26]. For this study, a modified version of the graphic used by Mekhora et al. [27] was applied.

b. Tergumed 530 series devices

The isometric maximum force measurements were performed on the Tergumed 530 series devices from the Proxomed Company (proxomed Medizintechnik GmbH, Alzenau, Germany). Each device can be individually adjusted to the patient through many adjustment options (e.g., seat and backrest height or distance to footrest). The measured force values were expressed in newton-metres (Nm).

c. MVN Link by Xsens

Inertial motion capture data were recorded using the motion analysis system MVN BIOMECH Link by Xsens (Xsens Technologies B.V., Enschede, The Netherlands). By means of 17 miniature inertial measurement units (“MTx”, dimensions: 3.5 cm × 2.5 cm × 0.8 cm), which contain 3D linear accelerometers, 3D magnetometers measuring the (Earth’s) magnetic field, 3D rate gyroscopes and a barometer, biomechanical data were obtained. Although the initial sampling rate was 240 Hz, we down-framed the data to 24 Hz since few, if any, high-speed movements occurred during the dental treatment measurements. In the related methodology article of the SOPEZ project [23], more detailed information can be found.

### 2.3. Assessment Protocol

- Pain intensity

By means of the Nordic Questionnaire’s VAS scales, values for the pain intensity were collected before each training unit. The subjects indicated how much pain they were currently experiencing for each body region. The regions assessed were the neck, the upper and lower back and the left and right shoulders, as these are the most affected segments in dentistry.

b. Maximum voluntary isometric contraction (MVIC)

At baseline and after finishing the intervention, the maximum isometric force was recorded for the trunk’s three planes of motion (sagittal, frontal and rotational plane). For data collection, the measurement was performed three times on each device and the mean value was calculated in order to minimise measurement errors. Each device was calibrated before each measurement. There was a pause of 45 s between measurements, which was specified by the software.

c. Ergonomic analysis of the working posture

In order to collect the biomechanical data that provide the information on the position and angle of the joints, the analysis was performed during a dental treatment simulation on a dummy head. For this purpose, the participants were paired in treatment teams, each consisting of a dentist and a dental assistant; they were then measured together simultaneously. The measurements were carried out within the treatment concept that was familiar to the dentist and dental assistant team. The treatment team was equipped with 17 inertial motion sensors from the MVN BIOMECH Link system from Xsens, which were attached to the subject via a lycra suit. To simulate the work situation, the participants were then assigned to a specifically designed work simulation according to their respective specialisation as generalists, oral and maxillofacial surgeons, endodontologists or orthodontists. The participants then carried out a predefined sequence of activities over a period of approximately 30 min whereby the defined steps were always announced and measured by the investigator shortly beforehand. The exact treatment protocol for each field of specialisation can be found in Ohlendorf et al. [19].

### 2.4. Intervention

**Figure 1:**
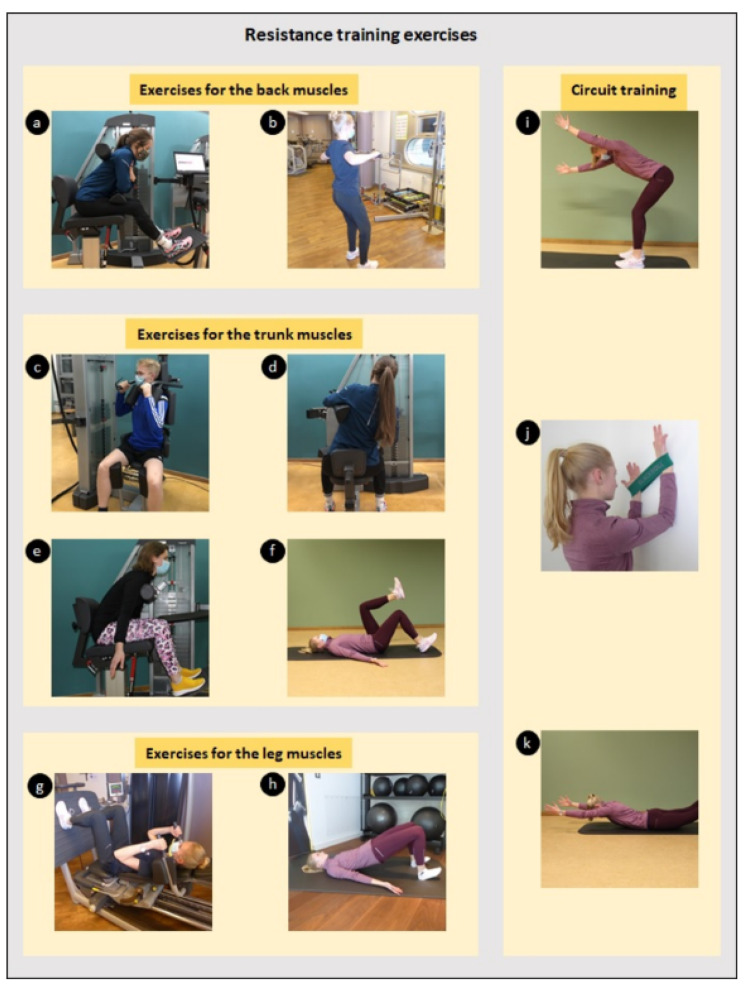
Exercises of the resistance training programme: (**a**) back extension, (**b**) wide rowing, (**c**) trunk rotation, (**d**) lateral flexion of the trunk, (**e**) trunk flexion, (**f**) dead bugs, (**g**) leg press, (**h**) glute bridge, (**i**) arm wiggles, (**j**) wall walk and (**k**) superman.

Exercises that can strengthen the stabilising postural muscles were selected for the training programme, especially exercises that target the musculature of the back, neck, shoulders and trunk. These correspond to the predominant regions for musculoskeletal complaints among dentists and dental assistants in the literature [1,4,6,28,29]. Each workout began with a five-minute warm-up session consisting of stair running. For the strength training of the back, trunk and leg muscles, exercises on machines and body-weight exercises were selected (Figure 1). Except for the circuit training exercises, which were performed at the end of each training session, all exercises were performed for 3 sets of 12 repetitions with a one-minute recovery break between each set.

In order to train the musculature of the back, the participants performed the “back extension” exercise on the machine and the “wide rowing” exercise on the cable tower (Figure 1a,b). For the trunk muscles, the participants performed the “trunk flexion”, “lateral flexion of the trunk” and “trunk rotation” exercises on the machine. In addition, the “dead bug” exercise was conducted (Figure 1c–f).

For the training of the legs, the participants performed the “leg press” and “glute bridge” exercises (Figure 1g,h). The intensity was controlled with weights placed on the stomach or hip. The order of the exercises was randomised.

At the end of the specific exercises, circuit training consisting of three exercises over three sets was performed. The exercise time for each exercise was 20–30 s, while the rest time was between 20 and 30 s. In order to increase the intensity, the exercise and break durations could be adjusted depending on the fitness level.

For the first circuit exercise, called “wall walking”, the participants stood close to a wall with the wrists in a closed latex band (loop), the palms facing the participants and with the forearms pulled at right angles; the arms were then moved up and down (Figure 1j). The intensity of the exercise could be influenced by the strength of the loop. For a second exercise—called “superman”—the participants lay on their stomachs on the floor with their arms stretched out in front (Figure 1k); the arms and legs were then raised and lowered simultaneously, with an increase in intensity achieved by lifting diagonally and increasing the time interval. For the final circuit exercise—called “arms wiggle”—the participants stood hip-width apart with knees bent and the upper body slightly forward (Figure 1i); the arms were extended and the participants alternately wiggled up and down with the arms, whereby the intensity could be increased by the use of weights and an increase in the time interval. The training intensity was chosen as follows:

(1) For the training in the devices (Figure 1a,c–e), 80% of the determined maximum strength was set as the training intensity.
(2) For all other training exercises (Figure 1b,f–h), the intensity was chosen so that 12 repetitions were possible with technically clean execution.

The intensity was increased once subjects could complete 12 repetitions effortlessly. The load control was somewhat different for circuit training (Figure 1i–k). Here, the load duration was increased as soon as the subjects were able to tolerate it.

The number of repetitions and the intensity of each participant’s exercise were noted for each training unit to ensure optimum load control.

### 2.5. Data Analysis

All recorded kinematic data were post-processed using the Xsens software. Subsequently, the joint angle data were further processed in custom written files with MATLAB^®^ vR2020a software (The Mathworks Inc., Natick, MA, USA). By means of a code developed by Maurer-Grubinger et al. [30], an ergonomic risk assessment using RULA was applied on the recorded kinematic data. Details on this script can be found in Maurer-Grubinger et al. [30]. For the current analysis, we chose the “relative time score” outcome value.

This outcome value was calculated from the relative portion of the time spent at each RULA score. The corresponding formula is shown below:

relative time spent at RULA score 1*1 + relative time spent at RULA score 2*2 + relative time spent at RULA score 3*3(..) + relative time spent at RULA score 7*7

This outcome value was then calculated for the following body regions:
Final Score - RULA Step 152.Neck Score - RULA Step 93.Trunk Score - RULA Step 104.Upper Arm Score (left and right) - RULA Step 15.Lower Arm Score (left and right) - RULA Step 26.Wrist Score    (left and right) - RULA Step 3 + 4

### 2.6. Statistical Analysis

The statistical data analysis was performed in Excel2016 (Microsoft Corporation, Redmond, WA, USA), MATLAB^®^ vR2020a (The Mathworks Inc., Natick, MA, USA) and SPSS Statistics 26 (IBM Deutschland GmbH, Ehningen, Germany). All data were tested for a normal distribution using the Shapiro–Wilk test. Except for the pain intensity data for each training unit, most data were normally distributed, thus, the median was calculated for the figures, thereby indicating the pain intensity at each training unit. To test for significant differences in pain intensity before and after the intervention, training units 1 and 2 and 19 and 20, respectively, were added in order to invalidate outliers. The Friedman test with multiple comparisons and a Bonferroni–Holm correction were also applied to the pain intensity of training units 1, 5, 10, 15 and 20. These tests were performed in order to identify an overall effect and the changes within the intervention of the 20 training units. For the outcome values of MVIC and RULA score, parametric tests were applied. In order to test for significant differences between baseline and post-intervention values, the dependent t-test was performed. The Pearson correlation coefficient was calculated between pain, force and posture data for both baseline and pre-post-comparison. The significance level was set at α = 5%.

## 3. Results

### 3.1. Pain Intensity

**Figure 2:**
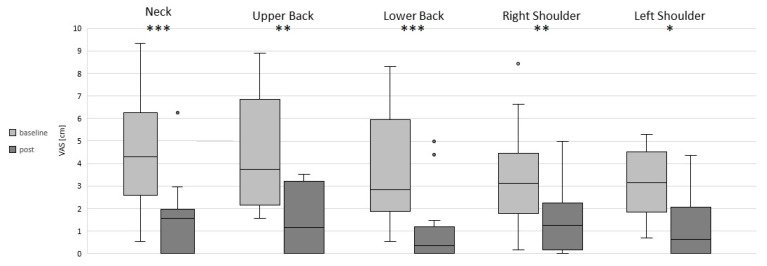
Pain intensity at baseline and post-intervention in the neck, upper back, lower back and right and left shoulders. Significant differences are marked with asterisks: * *p* < 0.05, ** *p* < 0.01 and *** *p* < 0.001.

The intervention showed significant effects for those participants who reported pain at baseline. Accordingly, the pain intensity significantly decreased after the intervention in the neck (*p* < 0.001), upper back (*p* = 0.004), lower back (*p* > 0.001) and in the right (*p* = 0.005) and left (*p* = 0.02) shoulders (Figure 2, Table 2). In addition, the dispersion of pain intensity was considerably reduced, as shown in the IQR (Table 2) as well as in the whiskers of the box plots (Figure 2). Here, especially in the neck and lower back, the dispersion was significantly reduced (Figure 2). The sample size varied depending on the region: neck n = 15, upper back n = 9, lower back n = 14, right shoulder n = 13 and left shoulder n = 10.

Considering the overall changes within the 20 training units, the neck (*p* = 0.001) and the upper (*p* = 0.012) and lower (*p* = 0.001) back showed significant effects (Table 3). The pain intensity of the neck was the first to be significantly reduced (*p* = 0.03), which occurred after five training units (Table 3). In the upper (*p* = 0.005) and lower (*p* = 0.038) back, the first significant reduction in pain intensity occurred after 10 training units (Table 3).

**Figure 3:**
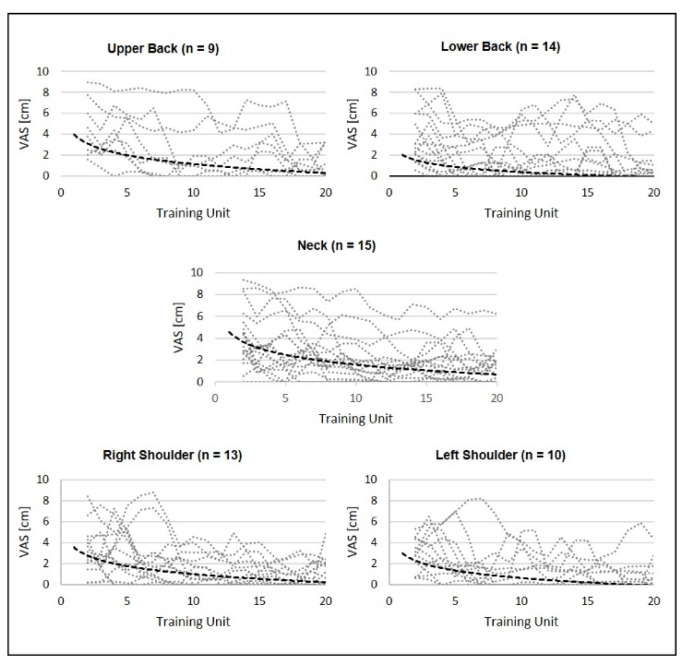
Exponential median and moving average over 20 training units for the upper back, lower back, neck and right and left shoulders. The dotted line indicates pain values for each subject. The respective sample size is indicated at each subfigure.

The individual pain intensity progression in the upper and lower back, neck and right and left shoulders over the 20 training units is illustrated in Figure 3. All five subfigures reveal a large scattering between the participants’ pain indications and the heterogeneous progression of the pain intensity. However, an overall trend in the reduction of pain intensity can be derived from each subfigure (Figure 3). This trend can also be seen at the individual level in all queried body regions. This shows that most subjects experienced only moderate to mild pain during the course of the intervention, whereas many experienced severe pain at baseline (Figure 3).

### 3.2. Maximum Isometric Force

**Figure 4:**
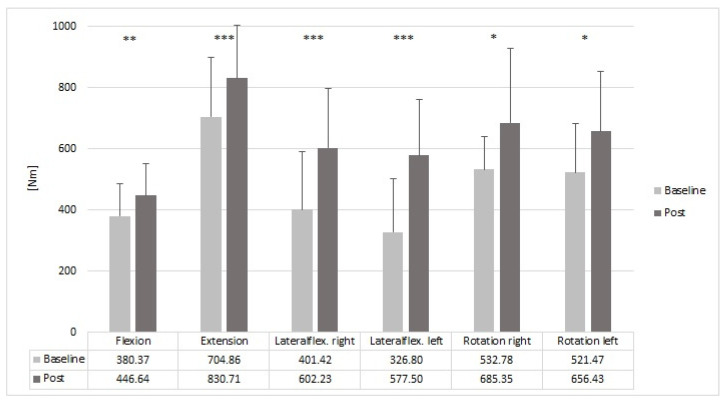
Effects of a 10-week resistance training programme on the MVIC of the trunk. The table shows the mean values of the MVIC at baseline and post-intervention for each outcome. Significant differences are marked with asterisks: * *p* < 0.05, ** *p* < 0.01 and *** *p* < 0.001.

The intervention had significant effects on the maximum isometric force in all tested movements (Figure 4). In the trunk flexion, participants increased their MVIC by 17.42% from 380.37 Nm to 446.64 Nm (*p* = 0.001). Comparable improvements were found for the trunk extension with an improvement of 17.85% from 704.86 Nm to 830.71 Nm (*p* = 0.004). While the lateral flexion showed almost a 20% difference at baseline between the right and left sides (r > l), the imbalance was reduced to less than 5% after intervention. The relative gains were clearly higher in the frontal plane than in the sagittal plane. For the right lateral flexion, participants increased their MVIC by 50.02% from 401.42 Nm to 602.27 Nm (*p* < 0.001) and for the left lateral flexion, participants increased their MVIC by 76.71% from 326.80 Nm to 577.50 Nm (*p* < 0.001). The upper body rotation showed less imbalance between both body sides at baseline and, likewise, showed similar improvements to both sides post-intervention, such as the forces in the sagittal plane. In the right rotation, the participants improved by 28.64% from 532.78 Nm to 685.36 Nm (*p* = 0.01) and in the left rotation, the participants improved by 25.88% from 521.47 Nm to 656.43 Nm (*p* = 0.011) (Figure 4).

### 3.3. Ergonomic Analysis of the Working Posture

**Figure 5:**
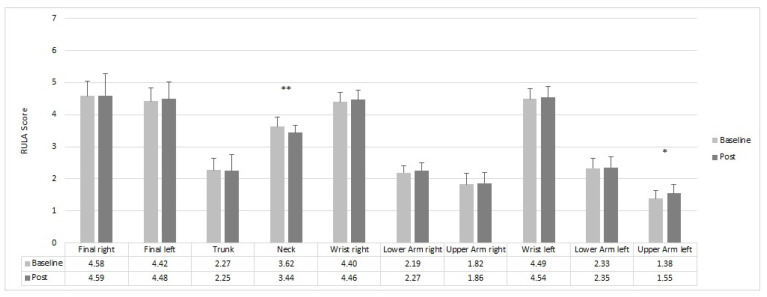
Effects of a 10-week resistance training on the body posture during dental treatment. Mean values for baseline and post-intervention for each body region are shown below. Significant differences are marked with asterisks: * *p* < 0.05 and ** *p* < 0.01.

The participants showed a marginal change in the ergonomic risk following the 10-week resistance training intervention. Only for the neck could a significant improvement (*p* = 0.02) of the ergonomic risk be found—going from 3.62 to 3.44. In contrast, a significant worsening (*p* = 0.018) of the ergonomic risk from 1.38 to 1.55 was found for the left upper arm score (Figure 5).

## 4. Discussion

In this study, for the first time, the effects of a systematic resistance training programme on the pain intensity and ergonomic risk experienced during dental treatments by dentists and dental assistants were investigated. Our findings demonstrated that resistance training as a behavioural prevention is a suitable measure in dentistry workplace health promotion. Firstly, the 10-week resistance training programme resulted in a significant increase in maximum strength in all tests (Figure 3). The relative improvements were about 50% (right) and 77% (left) for trunk lateral flexion, 29% (right) and 26% (left) for trunk rotation and 17% and 18% for trunk flexion and extension, respectively. In a similar study with industrial workers, a progressive resistance training programme led to an increase in the maximum strength in all tested body regions (biceps, triceps, deltoid, quadriceps femoris and hamstrings and triceps surae) [20]. Further results from previous studies that investigated the effects of resistance training on strength parameters are in line with our current findings [31,32].

The improvements in strength in the current study are associated with a reduction in pain at both the individual and statistical levels. The statistics revealed a significant decrease in pain intensity in the neck and lower back after the 5th training unit and in the upper back after the 10th training unit. The results indicate either a faster relief in pain in those regions that are most affected (Figure 2 and Figure 3, Table 2) or that the exercise selection is particularly effective in targeting the aforementioned body regions. Previous studies with a comparable sample size [22,33] have demonstrated a substantial reduction in neck and shoulder pain using resistance training. In addition, Andersen et al. [34] showed a significant decrease in pain in all investigated body regions in a one-year exercise intervention in office workers after physical exercising or resistance training.

It can be hypothesised that this increase in strength of the trunk may have led to an effective reduction in pain. Improving strength has been proven to be effective for preventing and reducing pain [31,35]. In addition to the aforementioned overall strength gains across all strength tests, the reduction in muscular imbalances may have played a critical role in pain reduction (Figure 3). The baseline results demonstrated a difference of almost 20% in the lateral flexion between the right and left side. This clear imbalance in the trunk may have also had an impact on pain intensity in the sample as dental work activity is characterised by biaxial movements. Thus, the reduction in muscular imbalance to less than 5% for lateral flexion may have had an impact on pain reduction, in addition to the overall increase in strength. This hypothesis can be supported by findings from the strength ratios of cervicothoracic extension and craniocervical flexion between females with and without idiopathic neck pain. Here, patients with idiopathic neck pain showed a reduced craniocervical flexion strength ratio compared to healthy subjects [36]. However, this increase in the strength of the trunk muscles, combined with the reduced pain of the trunk region, does not significantly affect dental work activities. The kinematic data analysis of all relevant joint angles using RULA shows that the ergonomic risk is significantly reduced in the neck but increased in the left upper arm. All other regions, as well as the final score, remain unchanged. However, when considering the values (neck: 3.62–3.44; left upper arm: 1.38–1.55) of the two significant parameters, their clinical significance is rather secondary or marginal. Even if the ergonomic risk does not change overall or in the individual regions, it can be speculated here that the increase in trunk strength (evidenced by an increase in maximum strength) enables the upper body to maintain or perform positions or movements more painlessly over a longer period of time. Since the increased force appears to have a significantly positive effect on pain intensity, resistance training is suitable as a behavioural preventive measure for the reduction of pain in dental professionals.

According to the current evidence on the effect of resistance training on pain in other professions [15,16,22], this study has proved that a systematic strength training programme for dental personnel (dentists and dental assistants) over the course of ten weeks, with exercises being performed twice a week for one hour, reduces the pain intensity significantly in all five recorded body regions and improves the MVIC values.

The only study that investigated comparable outcomes in dental personnel was a pilot study by Yiu et al. [17]. They found a significant improvement in the RULA scores, with an increase of 1.88 points, as well as improvement in the isometric maximal strength of the neck flexor and extensor muscles (17.2% and 23.2%, respectively), but no significant improvement in the isometric strength of the shoulder muscles and symptom severity at the neck/shoulder region was recorded. In contrast, we measured dentists and dental assistants who already had more professional experience and, therefore, a higher pain prevalence, which has been shown in a Germany-wide survey among dentists [6] and dental assistants [1]. This fact may explain why the symptom severity did not decrease in the dental students of Yiu et al.’s study [17]. One advantage of the present study is the permanent supervision or instruction of all participants in each training session by sports scientists. Appropriate guidance of the subjects seems to have a positive influence on the results of resistance training interventions since adequate coaching ensures proper loading of the muscles in addition to motivation.

However, there are also limitations to be mentioned in this study. In the present study, the MVIC values of the trunk extension, flexion, lateral flexion and rotation were measured. However, the strength developed in the shoulder and neck muscles could not be measured; it can only be hypothesised that the strength in this region had, likewise, also improved. Horizontal rowing on the pulley or wall walking with the mini-band explicitly strengthened the muscles of the upper back and posterior shoulder, as well as the external rotators of the shoulder. Furthermore, the training programme was not based on a previously implemented programme but was planned and designed by experienced sports scientists in the field, thereby making it methodologically difficult to compare with similar strength training interventions in other professions. For example, a circuit of three exercises was incorporated in which the load control was based on time rather than repetition, while the set numbers and resistance increased as in the other exercises.

It is important to address the unequal gender distribution of the study group (3 males and 14 females). In light of the fact that women report pain more frequently and more intensely than men, it cannot be excluded that the effects of strength training on pain intensity would equally occur in a higher proportion of men. A possible consequence of this could be a reduced effect due to a reduced pain perception.

The missing dental control group in the study was due to the health situation in Germany in the summer of 2020 when the training was scheduled to be conducted. Originally, a control group was planned that would also undergo the same training programme as the intervention group following pre- and post-measurements (with a break of 12 weeks). The lack of a control group has a serious impact on the quality of the study. A confounding of the intervention effect with the effect of other occurring factors cannot be excluded and, in the worst case, the effect is erroneously attributed to the intervention. Nevertheless, the demonstrated effects of strength training on reducing pain intensity are high. It is hardly to be expected that such effects would be equalized by a potential control group, but of course it cannot be excluded.

Unfortunately, the SARS-CoV-19 virus, on the one hand, led to a lockdown and, consequently, the physiotherapy practice in which the training was carried out was affected by special hygiene and opening-hours conditions. On the other hand, many volunteers cancelled their participation in the study during this period because they were afraid for their professional existence. In this context, they wished to minimise exposing their health to COVID risk factors as much as possible. Therefore, priority was given to the completion of the intervention group. However, this group was also impacted by the pandemic with a shortening of the intervention duration from 12 to 10 weeks. A further limiting factor was the low sample size for the pain analysis of some regions (e.g., upper back and left shoulder). Since the research question is pain reduction, only those subjects who reported pain in one of the first two training units were included for the sub-analysis. Furthermore, it should be taken into account that the influence of the previous working day could also be possibly reflected in the evaluation of the complaints (VAS).

Thus, in summary, it can be concluded that strength training can be successfully used as a (tertiary) preventive measure to reduce/delay MSD. Basically, chronic complaints are a sign of overload, which can be reflected in MSD, e.g., in the lower back. In this context, active therapy approaches are more effective than, for example, passive physiotherapeutic measures [37].

Due to the high prevalence of MSD among dental personnel [1,4,6,9,29,38,39], further research is absolutely necessary. Since previous studies [14,40,41] have shown that the preventive re-design of the dental workplace has little positive effect on the working posture and since this study can prove that resistance training can subjectively reduce pain, further behavioural preventive measures, such as ergonomic training, stretch training or the combination of strength training and stretch training, should be analysed.

## 5. Conclusions

A 10-week resistance training programme for dentists and dental assistants had significant effects on pain intensity reduction and on the muscle strength of the trunk musculature, but a lesser effect on the ergonomic risk. Nevertheless, it is suitable as a behavioural preventive measure in order to reduce MSD in dentistry. The effects of behavioural preventive measures, such as ergonomic training, stretch training or a combination of resistance and stretch training, on pain intensity and ergonomic risk should be investigated in future studies. Furthermore, it should be investigated whether resistance training can serve as a (job-related) rehabilitative measure for re-integration into everyday working life.

## Figures and Tables

**Table 1:** Baseline characteristics of the participants by sex. Mean values with standard deviation (SD) in brackets are shown for the total characteristic group, and females and males. BMI indicates the body mass index.

	Total n = 17	Females n = 14	Males n = 3
Age [years]	35.71 (12.38)	32.14 (9.10)	52.33 (13.61)
Height [cm]	169.29 (6.31)	167.93 (5.69)	175.67 (5.86)
Weight [kg]	62.94 (8.69)	60.21 (6.59)	75.67 (5.13)
BMI [kg/m^2^]	21.89 (2.15)	21.33 (1.92)	24.51 (0.74)

**Table 2:** Descriptive data of the pain intensity at baseline and post-intervention in the neck, upper back, lower back and right and left shoulders.

	Neck		Upper Back		Lower Back		Right Shoulder		Left Shoulder	
	Pre	Post	Pre	Post	Pre	Post	Pre	Post	Pre	Post
median	4.295	1.565	3.75	1.175	2.853	0.343	3.125	1.25	3.163	0.625
IQR	3.67	1.955	4.69	3.205	4.065	1.193	2.697	2.054	2.655	2.108
*p*-value	<0.001		0.004		<0.001		0.005		0.02	

**Table 3:** Results for the first significance of pain reduction. Friedman test with multiple comparisons between the 1st, 5th, 10th, 15th and 20th training units was applied. The row “1st significance” indicates the first significant comparison obtained between the first and subsequent training units. * Bonferroni–Holm correction was applied on multiple comparison calculations.

	Neck	Upper Back	Lower Back	Right Shoulder	Left Shoulder
Friedman’s Chi^2^	0.001	0.012	0.001	0.362	0.258
1st significance	5	10	5	-	-
*p*-value *	0.030	0.005	0.038	-	-

## Data Availability

All data generated or analysed during this study are included in this published article.

## Supplementary Materials

The following supporting information can be downloaded at: https://www.mdpi.com/article/10.3390/s22208069/s1. Figure S1. The pain intensity on a scale of no pain intensity to maximum pain intensity.

## Abbreviations

MSD	musculoskeletal disorders
RULA	rapid upper limb assessment
SOPEZ	study for the optimization of ergonomics in the dental practice
SD	standard deviation
VAS	visual analogue scale
MVIC	maximum voluntary isometric contraction
