# Supplementary figures and images for: Effects of Resistance Training as a Behavioural Preventive Measure on Musculoskeletal Complaints, Maximum Strength and Ergonomic Risk in Dentists and Dental Assistants
Source: Sensors (Basel). 2022 Oct 21;22(20):8069. doi: 10.3390/s22208069 (PMC9609802; doi:10.3390/s22208069)

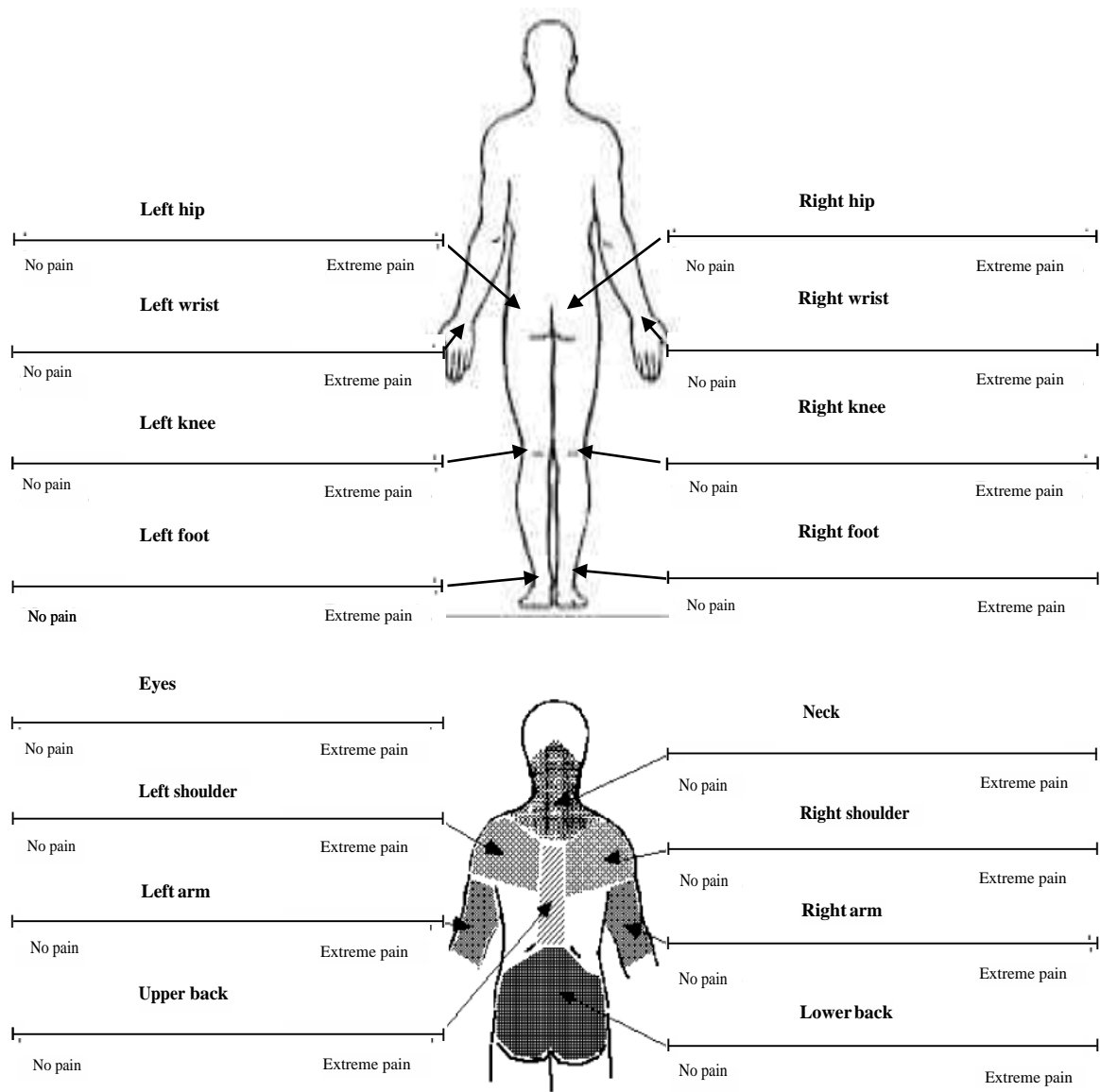

Figure S1. The pain intensity on a scale of no pain intensity to maximum pain intensity.

Supplement: Supplementary file 1 [file sensors-22-08069-s001.zip › sensors-1908401-supplementary.pdf]
